# Sample illumination device facilitates in situ light-coupled NMR spectroscopy without fibre optics

**DOI:** 10.1038/s42004-022-00704-5

**Published:** 2022-08-04

**Authors:** Jack E. Bramham, Alexander P. Golovanov

**Affiliations:** Department of Chemistry, School of Natural Sciences, Faculty of Science and Engineering, The University of Manchester, Manchester, M13 9PL UK

**Keywords:** Solution-state NMR, Physical chemistry, Solution-state NMR, Photochemistry

## Abstract

In situ illumination of liquid-state nuclear magnetic resonance (NMR) samples makes it possible for a wide range of light-dependent chemical and biological phenomena to be studied by the powerful analytical technique. However, the position of an NMR sample deep within the bore of the spectrometer magnet renders such illumination challenging. Here, we demonstrate the working principles of a sample illumination device (NMRtorch) where a lighthead containing an LED array is positioned directly at the top of an NMRtorch tube which is inserted into the NMR spectrometer. The wall of the tube itself acts as a light guide, illuminating the sample from the outside. We explore how this new setup performs in a number of photo-NMR applications, including photoisomerisation and photo-chemically induced dynamic nuclear polarisation (photo-CIDNP), and demonstrate the potential for ultraviolet (UV) degradation studies with continuous online NMR assessment. This setup enables users of any typical liquid-state spectrometer to easily perform in situ photo-NMR experiments, using a wide range of wavelengths.

## Abstract (web summary)

In situ illumination of liquid-state NMR samples allows to characterise light-dependent chemical and biological phenomena, but, in practice, the position of an NMR sample deep within the bore of a spectrometer magnet renders such illumination challenging. Here, the authors demonstrate the working principles of a sample illumination device, with an LED array positioned directly at the top of special sample tube, which is inserted into the NMR spectrometer.

## Introduction

Light is critical for a myriad of chemical and biological reactions. Ultraviolet (UV), visible, and infrared (IR) light can act as ideal triggers to connect our macroscopic world with the nanoscale or molecular world, for example, to modulate material properties^1,2^, molecular switches^3^, gene expression^4^, targeted drug release^5,6^, enzymatic reactions^7–9^, and many other physical processes. The ability to incorporate controlled light illumination into experimental and analytical procedures is therefore essential.

Nuclear magnetic resonance (NMR) spectroscopy is widely used to study the properties and behaviour of molecules, reactions and materials^10^. Given the capabilities of NMR spectroscopy, a number of approaches to illuminate NMR samples have been developed (recently comprehensively reviewed by Nitschke et al.^11^ and Ji et al.^12^), allowing a number of light-sensitive systems, including photocatalysed reactions^13,14^ and polymerisation^15,16^, to be studied by light-coupled NMR spectroscopy (often simply referred to as photo-NMR). Furthermore, illumination may be directly beneficial for some NMR applications; for example, by dramatically improving signal intensity through photo-chemically induced dynamic nuclear polarisation (photo-CIDNP), enabling the detection of nanomolar quantities of biomolecules^17–19^, incorporating into flow probes^20,21^, or allowing studies of proteins^22–24^ or chemical reactions^25^.

Direct in situ illumination of samples inside the NMR spectrometer, coinciding with spectral acquisition, is largely preferable to ex situ illumination^11^. However, the position of an NMR sample deep within the magnet bore of a typical NMR spectrometer makes it technically challenging to deliver sufficient levels of light to uniformly illuminate the sample. Historically, external LASERs guided by mirrors, glass rods or optical fibres have been used^26–28^, but such light sources are relatively expensive and prone to intense localised sample heating, with a fairly limited variety of wavelengths usually available in a specific lab^11^. Furthermore, open-beam systems with mirrors may require complex adjustments and additional safety measures, while systems using optical fibres may suffer from losses of light^29^. Moreover, such fibres and associated inserts may hinder magnetic field homogeneity and shimming, and can be generally difficult to work with, leading to low sample throughput. Recently, the rapid development and availability of inexpensive and powerful light-emitting diodes (LEDs) with a wide choice of emission wavelengths has led to their increasing use as light sources in photo-NMR^12,13,30–33^. Although NMR probe modifications to incorporate LED illumination directly inside the probehead have been suggested^27,34^, optical fibres (either alone or inside a coaxial insert) are still typically used to guide light from the LED positioned outside the NMR spectrometer to the NMR sample inside the magnet. However, these setups also suffer from the same disadvantages inherent to the LASER-fibre optic system, with the additional difficulty of coupling poorly collimated light from an LED into a thin optical fibre, leading to significant light intensity losses^12,30^.

In this work, we introduce an alternative universal strategy for delivering light generated by LEDs to typical solution-state NMR samples, without the need for fibre optics, or probehead modifications. In our design (which we named NMRtorch), one or more LEDs are mounted in a lighthead attached directly to the top of an NMRtorch tube, with this assembly inserted into the NMR magnet bore. The wall of the tube itself acts as a light guide, with light scattered only around the sample volume by special etchings covering this area, resulting in uniform illumination from the *outside* of the sample.

Here, we demonstrate the potential and capability of this new in situ photo-NMR approach to study a wide variety of phenomena. Firstly, we demonstrate very significant signal enhancements obtained in photo-CIDNP experiments, and show how light uniformity and intensity inside the NMR samples can be assessed, and optimised through positioning of light-scattering etchings. We show that several LEDs of different colours can be used in the same experiment, with complete NMR spectrometer control alongside RF pulses, e.g., to study the behaviour of photoswitches or to measure the light intensity directly inside the sample. We also show that our approach enables the delivery of significant UV radiation dosage within a short period of time, thus making photostability studies of chemicals and pharmaceuticals with simultaneous NMR detection possible. The convenience of using these design principles is expected to make photo-NMR applications more accessible on a wide range of old and new NMR spectrometers and probeheads.

## Results

### Principles of the NMRtorch design

**Fig. 1:**
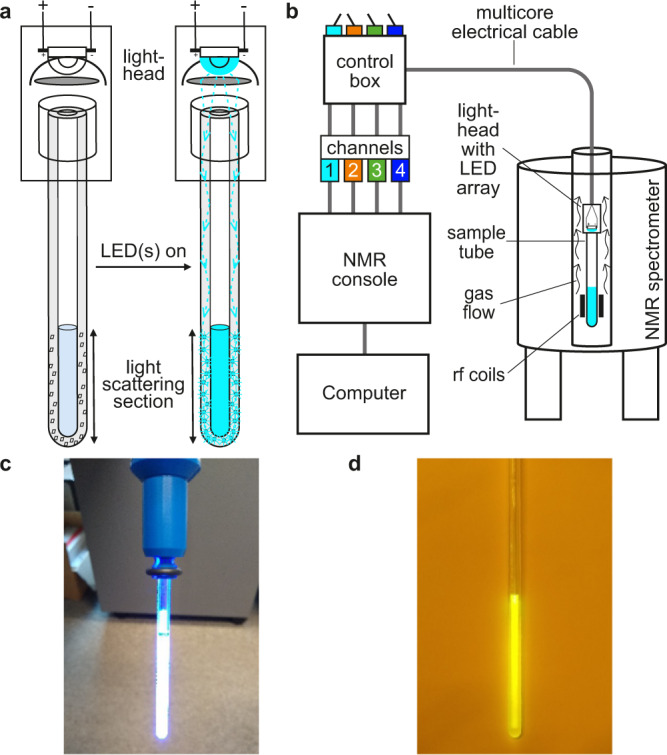
Principles of the NMRtorch. **a** Principle of the NMRtorch approach, with the NMR sample tube acting as a light guide. **b** Schematic of the NMRtorch setup. Not to scale. **c** Appearance under ambient light on an example NMRtorch tube filled with a typical sample and illuminated with 3 W 460 nm blue LED. Scattered light appears as white due to oversaturation of the photoimaging sensor. **d** Image of saturated solution of fluorescein sample illuminated by blue light, taken under ambient light through orange filter. The vibrant yellow fluorescence is visibly uniform across the sample.

In our proposed approach to illuminating NMR samples in situ, a lighthead (Fig. 1) containing one or more LEDs is positioned directly at the top of an NMRtorch tube, allowing light to enter the walls of the glass tube through the rim. The lighthead can be either attached to the tube, or can be positioned at the top after the sample is inserted. If used, the tube cap should be sufficiently optically clear (Fig. 1a). Optional optical elements, such as lenses or condensers, may be used to further focus light, although we found these to be of little benefit given the close proximity of the LED emitter to the top rim of the tube. A close positioning ensures that light does not diverge significantly before entering the tube. The NMR tube itself acts as a light guide, with light confined within the optically transparent wall of the tube, and propagated towards the sample area. In NMRtorch tubes, the glass around the sample volume at the bottom of the tube is modified by the introduction of light-scattering centres (achieved here by manual abrasive etching of the exterior surface). When the LEDs are illuminated, light is scattered here in all directions, thus preferentially illuminating the sample area from the outside (Fig. 1a). Following these modifications, standard heavy wall borosilicate or quartz 5 mm tubes with ~1.2–1.4 mm wall thickness worked well as prototypes in our experience.

The lighthead is connected by a multicore electrical cable to an electrical control box containing constant-current power supplies (Supplementary Fig. 1), with their triggers in turn connected to the NMR console controlled by a computer (Fig. 1b). Furthermore, in the NMRtorch setup described here, four separate channels connect to the NMR console, allowing the use of multi-channel LED arrays, to maximise light output at a single wavelength, or to provide illumination at different wavelengths. In the current implementation, the NMR tube and attached lighthead are manually lowered into the spectrometer bore by the multicore electrical cable. Gas flow that is typically present in the magnet bore provides sufficient cooling for LEDs with lower power consumption (≤3 W) or under dimmed light operation. However, for more powerful LED arrays at constant illumination, a supplementary compressed gas line to the lighthead provides additional cooling (such as used here with 10 W UV lighthead), with temperature monitored by an optional temperature sensor in the lighthead. The proposed arrangement enables convenient delivery of high-intensity light (Fig. 1c) to the sample area achieving high uniformity (Fig. 1d), while essentially allowing the user to handle the NMR sample tube as normal, with easy tube filling and capping, avoiding any inserts or additional sources of magnetic field inhomogeneity in the sample area. Filling and capping the tubes can be done in the glovebox, thus allowing to work with oxygen and moisture-sensitive samples. The etchings on the outside surface of NMRtorch tubes, in our experience, do not affect NMR tuning, shimming, or water suppression, and no significant effects on the signal lineshape were observed, while allowing easy washing of sample tubes. Furthermore, as all electrical components in the lighthead are positioned sufficiently far away from the NMR detection region, we have not noticed any signs of electromagnetic interference caused by LED switching.

### Using NMRtorch for photo-CIDNP experiments

**Fig. 2:**
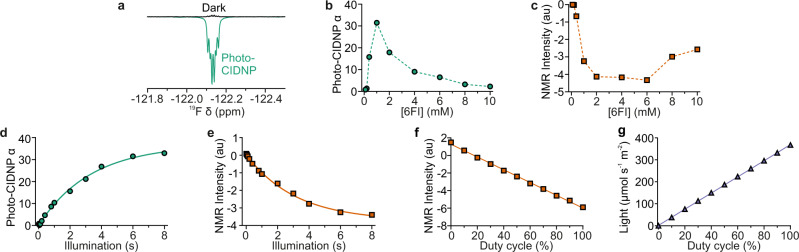
Photo-CIDNP effect observed for 6FI molecule using the NMRtorch apparatus. **a** Maximum observed 6FI photo-CIDNP effect, with 1 mM 6FI and 0.4 mM FMN, following 6 s illumination with 460 nm light. Both dark and illuminated photo-CIDNP spectra were acquired with one scan, and are shown here overlaid. Effect of 6FI concentration (in the presence of 0.2 mM FMN) on **b** ^19^F photo-CIDNP enhancement factor α and **c** NMR signal intensity, with 6 s illumination time. Effect of illumination time on **d** photo-CIDNP α and **e** signal intensity of 1 mM 6FI with 0.2 mM FMN, with exponential fits. **f** Effect of LED PWM (100 Hz) duty cycle on relative intensity of 6FI signal (at 6 mM, with 0.2 mM FMN present) recorded with 6 s illumination time. **g** The corresponding light intensity measured in PPFD units ex situ at the side of the sample with a photometer is also shown. The experimental data show linear dependence on duty cycle. All experiments recorded with single blue LED (460 nm, 3 W).

Photo-chemically induced dynamic nuclear polarisation (photo-CIDNP), where observed NMR signal intensities of aromatic compounds in the presence of a photosensitiser are modulated upon illumination, has been previously demonstrated using illumination with LASERs^18,19,22,35^, and more recently, with LEDs^13,32,33^. Here, we explored the suitability and effectiveness of NMRtorch for such photo-CIDNP experiments, by recording ^19^F NMR spectra of 6-fluoroindole (6FI) with photosensitisation by flavin mononucleotide (FMN) under blue light illumination. With the NMRtorch setup in a 11.7T spectrometer, 6FI exhibited emissive photo-CIDNP, with negative ^19^F peaks upon illumination and a maximum 64-fold enhancement achieved with the lighthead containing a single LED with nominal 460 nm peak emission, 3 W power consumption (Fig. 2a). To our knowledge, this enhancement is the largest ever reported for ^19^F, which is remarkable given that no attempts were made here to remove oxygen from the samples, or introduce any other measures to prevent dye quenching. Convenient working with multiple samples allowed us to explore the concentration-dependence of photo-CIDNP effects. At 0.2 mM FMN, the highest photo-CIDNP enhancements (α, Eq. 1) were observed at 1 mM 6FI, with lower enhancements observed at both higher and lower concentrations (Fig. 2b). As the highest absolute signal intensity for illuminated samples was observed at 6 mM 6FI (Fig. 2c), this concentration was chosen for further NMR imaging experiments where absolute signal intensity is critical. Photo-CIDNP enhancements and absolute signal intensities were also observed to increase with illumination time, yet began to plateau at values above 6 s (Fig. 2d, e). We found that the easy process of sample tube filling, capping and attaching to the NMRtorch lighthead enabled multiple photo-CIDNP samples to be run efficiently and consistently.

As light intensity is an important experimental parameter, it needs to be controlled and measured in the sample area. LED brightness can be routinely dimmed by controlling duty cycle (i.e., the fraction of time when the light is “on” during repeating short time periods) through pulse width modulation (PWM). Here, we incorporated this PWM control into the NMR pulse sequence, such that light intensity (between 0 and 100% duty cycle) before RF pulses was controlled directly by the NMR console (see Supplementary Software 1). Both the ^19^F NMR photo-CIDNP effect for 6FI (Fig. 2f), and the light intensity measured on the surface of the sample area using a photometer (Fig. 2g, with the assumption that similar amount of light should be scattered by the surface towards the inside area), were observed to be linear with LED duty cycle. This magnitude of 6FI photo-CIDNP effect and its linearity with increasing light intensity means, first, that the light gets inside the sample efficiently and second, this parameter related to photo-CIDNP effect can be used to assess the uniformity of light distribution across the length of the NMR sample in NMR imaging experiments. Although photo-CIDNP has previously been used to assess the effectiveness of sample illumination approaches^30^ and light distribution uniformity^29^, here, to account for non-linearity of magnetic field gradients at the edges of NMR-active volume, we propose the use of position-dependant photo-CIDNP enhancement factors, rather than raw photo-CIDNP spectra^29^ obtained in imaging experiments. As the photo-CIDNP signal intensities and enhancement factors are directly proportional to light intensity, the profiles formed by position-dependent enhancement factors (${\alpha }_{{{{{{\rm{Z}}}}}}}$, defined by Eq. 3) report directly on light intensity distribution inside the sample, along *Z*-axis.

**Fig. 3:**
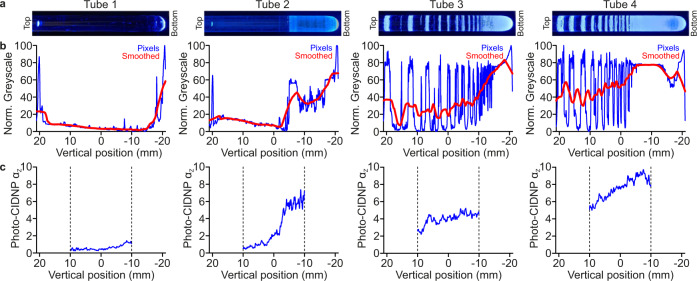
Effect of tube etching patterns on light distribution in NMRtorch sample tubes along the *Z*-axis. Tube 1—unetched; Tube 2—only bottom portion etched; Tubes 3 and 4—etched in a similar pattern, with tube 4 etched more than tube 3. **a** Photo images of example tubes illuminated with attenuated 460 nm light to avoid detector saturation. **b** Pixel brightness analysis of tube images, with pixel greyscale values normalised between lowest and highest values for each image as a measure of scattered light intensity. Smoothed values calculated using moving averages over ±2.5 mm. The tubes in **a** and **b** were filled with H_2_O to a depth of 40 mm from tube bottom. **c** NMR assessment of light distribution based on position-dependent photo-CIDNP enhancement (${\alpha }_{{{{{{\rm{Z}}}}}}}$) of 6 mM 6FI + 0.2 mM FMN in NMR imaging experiments with 6 s illumination times. Dashed vertical lines indicate the extent of the NMR-detectable volume. Vertical position measured along the *Z*-axis of the sample (here, horizontal), relative to the centre of the NMR coil, with the positive numbers towards the top of the tube.

In the NMRtorch setup, the positioning of light-scattering centres (created here by etching the outside surface of the tube with abrasive diamond files and sandpaper, using a simple lath) controls light distribution and intensity (Fig. 3). Excessive etchings at the top of the sample may cause the majority of light to be scattered at the top, with less light reaching the bottom of the sample area. We therefore propose that etchings should be distributed non-uniformly across the sample length, to achieve more uniform light distribution. To demonstrate the effect of the positioning of such etchings, light distribution in an illustrative set of NMRtorch tubes with different etching patterns was assessed along *Z*-axis by both pixel brightness analysis of photographic images, and photo-CIDNP position-dependent enhancement factors ${\alpha }_{{{{{{\rm{Z}}}}}}}$.

Here, smoothed pixel brightness characterisations of light distribution (Fig. 3b, with ±2.5 mm moving averages) were in good agreement with the observed photo-CIDNP position-dependent enhancements across the NMR-active volume (Fig. 3c). In the control unetched Tube 1, scattering was primarily observed at the sample meniscus and tube bottom, resulting in poor sample illumination and weak photo-CIDNP effects. Conversely, in sample tubes with various etched patterns (Tubes 2–4), light-scattering results in greater sample illumination and thus greater position-dependent signal enhancement (${\alpha }_{{{{{{\rm{Z}}}}}}}$). This distribution of etchings can be used to guide light distribution in the sample: for example, in Tube 2, the etched bottom portion experiences greater sample illumination than the unetched top portion of the sample region, whereas adding etchings in this area, in Tubes 3 and 4, increased illumination in the upper portion. Additionally, the degree of etching can be used to control overall illumination intensity, with the more frequent etchings on Tube 4 resulting in greater light intensity (averaging 260.9 ± 44.9 µmol m^−2^ s^−1^ across the sample area) and photo-CIDNP enhancements than Tube 3 (117.2 ± 33.3 µmol m^−2^ s^−1^). Notably, in the illustrative Tubes 2–4 shown here, light was more intense at the bottom of the sample, further away from the LED, where the etchings were denser. These experiments show that by controlling the positioning of the etchings it is possible to control the light distribution around the sample area of the NMRtorch tube, with the position-dependent photo-CIDNP signal enhancement parameter ${\alpha }_{{{{{{\rm{Z}}}}}}}$ being a convenient indicator of both the uniformity and overall light intensity in the sample area along *Z*-axis. It should be noted that due to efficient averaging of light scattered on the outside surface, and this surface acting essentially as an extended (planar) diffuse light source wrapped around the sample area, light inside the sample tube should be largely uniform in *X*–*Y* plane (Supplementary Fig. 2). For the following experiments we manually manufactured (using sandpaper and/or abrasive diamond tools) a collection of non-uniformly scratched tubes with sufficiently uniform light distribution along the whole sample length (Supplementary Fig. 3).

### Measuring light intensity using NMR actinometry

**Fig. 4:**
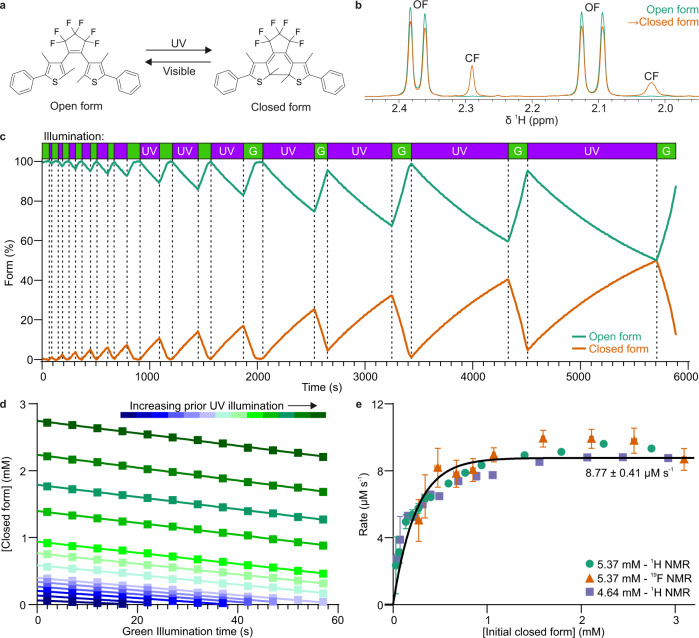
Actinometry using reversible in situ photoisomerisation of DAE with two LEDs. **a** Schematic of DAE photoisomerisation, with the observed actinometric reaction being the conversion from closed (CF) to open form (OF) under visible light. **b** Appearance of characteristic OF and CF DAE ^1^H NMR signals. **c** DAE photoisomerisation assessed by ^1^H NMR under repeated cycles of UV (280 nm) and lime green (550 nm) light illumination. **d** Determination of initial rates of isomerisation by linear fits to DAE CF concentration under green light illumination, with increasing prior UV illumination. **e** Initial rates of DAE photoisomerisation under green light derived from ^1^H and ^19^F NMR spectroscopy. The error bars and estimated fitting errors represent 95% confidence intervals.

To assess light intensity reaching the sample, we employed a molecular actinometer based on diarylethene (DAE)^36–38^, for which the closed (CF) to open form (OF) photocyclisation under visible light with known quantum yield can be used to determine photon flux, while the reverse OF → CF transition can be triggered by UV light to set the initial concentration of CF (Fig. 4a). For most actinometry approaches, numerous different concentration samples need to be prepared, and the conversion rates measured for each, to derive light intensity. However, here, by using an actinometer, such as DAE, with a stable photoreversible transition and a lighthead with two separate LEDs for visible (lime green) and UV light (here, 550 and 280 nm, respectively) light intensity can be determined from a single sample, in a single run. DAE CF concentration was varied by illumination with UV for a given time period before starting the actinometric CF → OF conversion with visible light (Fig. 4c), with simultaneous observation and determination of isomer concentration from characteristic signal integrals by either ^1^H (Fig. 4b) or ^19^F NMR (Supplementary Fig. 4). Furthermore, cycling between illumination wavelengths was directly incorporated into the pseudo-2D NMR pulse programme, enabling efficient and time accurate actinometric measurements.

Plotting the rates of the visible photoreaction (derived from linear fits under 550 nm illumination, Fig. 4d) vs initial CF concentration yields data that can be fitted to the model of Ji et al.^39^ (Eq. 4) to extract light intensity in the sample (Fig. 4e, see Supplementary Methods for further details). Here, the determined light intensity inside the sample was 438 ± 21 µEinstein L^−1^ s^−1^, corresponding to a photon flux density (PFD) of 251 ± 12 µmol m^−2^ s^−1^ at the surface of the sample. Perhaps as a useful reference, equivalent illumination would be achieved if the same sample was spread evenly over a flat area at a depth of 0.116 cm and directly illuminated with the same 550 nm LED from a distance of ~5 cm.

### Studying chemical photostability under UV illumination

We next tested whether the NMRtorch setup could deliver high-intensity UV light to the NMR sample. Studies of photoreactions, or photodegradation of pharmaceutical and biopharmaceutical products, would both benefit from simultaneous online observation of changes in NMR spectra under very intense light. The irradiation dosage typically required for stability testing of pharmaceutical products is defined in the International Council for Harmonisation of Technical Requirements for Pharmaceuticals for Human Use (ICH) Guidelines Q1B^40,41^, and suggests the use of 2% (w/v) quinine hydrochloride as an actinometric system, with prescribed dosage corresponding to greater than 0.5 increase in light absorbance at 400 nm following the UV-A exposure. In practice, such dosage is typically achieved after several hours in specialist UV illumination chambers, which do not allow easy online monitoring of the photodegradation process.

**Fig. 5:**
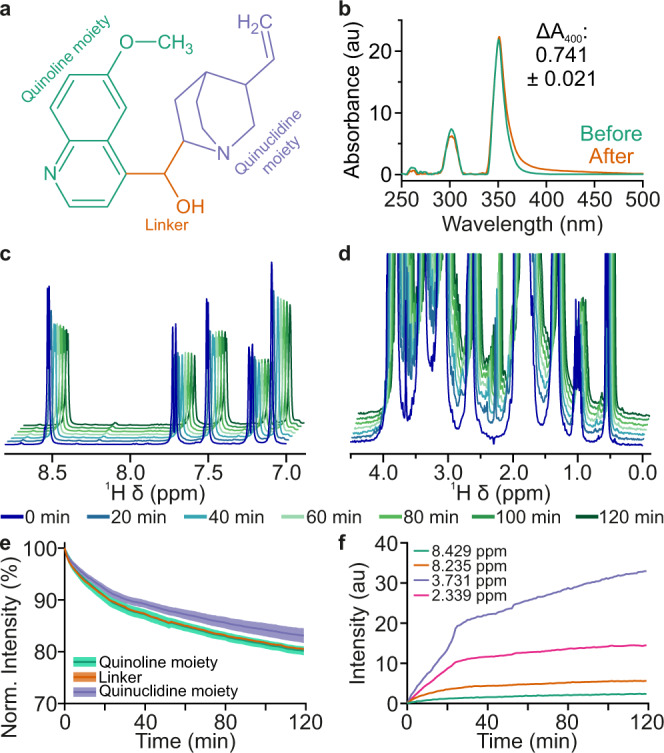
Photodegradation of quinine by UV irradiation using 10 W LED array with peak emission at 365 nm. **a** Chemical structure of quinine, with chemical moieties indicated. **b** UV/Vis absorbance spectrum of quinine before and after 2 h UV irradiation in NMRtorch tube. **c** Reduction in ^1^H NMR signals from quinoline moiety upon in situ UV illumination, monitored continuously. **d** Changes in aliphatic ^1^H NMR signals, including appearance of new peaks and spectral broadening. **e** Quinine signal intensity reduction over time, with each signal normalised against initial value. Shaded regions represent parameter error estimates with 95% confidence intervals. **f** Growth of degradation product signals over time.

Here, we explored whether the NMRtorch setup can deliver such a UV dosage within reasonable timeframe while allowing monitoring of sample degradation by ^1^H NMR spectra. A lighthead housing a 10 W UV LED array (365 nm peak wavelength) was employed and the degradation of quinine hydrochloride was simultaneously monitored. Even when a borosilicate glass tube was used, NMR spectral changes were readily observed within minutes (Fig. 5 and Supplementary Fig. 5), with change in optical density at 400 nm reaching 0.74 ± 0.02 after only 2 h irradiation (Fig. 5b), in line with ICH Q1B guidelines. Using a quartz tube instead of a borosilicate one enhanced the degradation rate by around three-fold (Supplementary Fig. 6). In the ^1^H NMR spectra acquired during UV irradiation of quinine, a series of phenomena were observed. Firstly, the intensity of the intact quinine peaks decreased (Fig. 5c) over time, with this occurring over two distinct timescales, and the greatest changes happening over the first 20 min (Fig. 5e). Monitoring signal reduction from different chemical moieties of the molecule allows to assess site-specific rates of degradation, with the quinoline moiety exhibiting greater reductions in signal intensity than the quinuclidine moiety. Secondly, significant spectral broadening was observed, particularly in the aliphatic region (Fig. 5d). Finally, a number of additional spectral peaks appear, with this occurring on a similar timescale to the reductions in the intact quinine peaks (Fig. 5d, f). Therefore, NMRtorch setup enables high-intensity irradiation of NMR samples in situ with UV light, achieving the doses prescribed by the regulatory guidelines within 2 h of experimental time, while allowing live monitoring of photodegradation.

### Studying multi-colour triggering of photoswitches

**Fig. 6:**
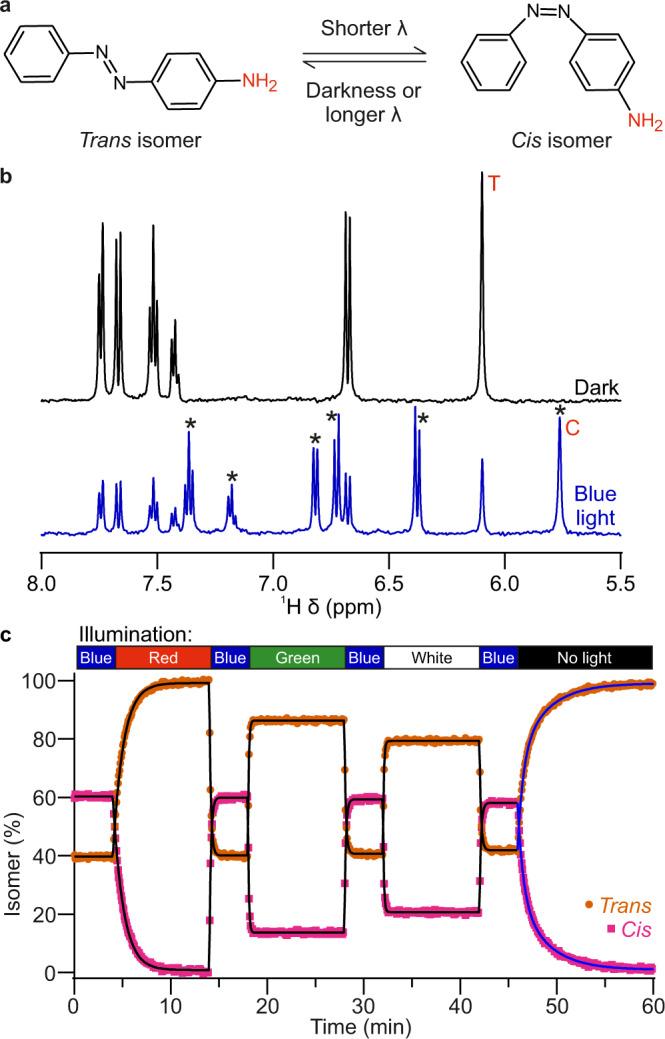
Photoisomerisation of 4-aminoazobenzene (AAB) studied using NMRtorch setup. **a** Schematic of *trans*–*cis* isomerisation of AAB, with amine groups used for NMR isomer assessments highlighted (red). **b** Example ^1^H NMR spectra at equilibrium under darkness (top) and blue light (bottom). Under darkness all signals arise from *trans* isomer, while asterisks denote upfield signals arising from *cis* isomer appearing under blue light. Intensity of equivalent amine NMR signals marked with T (*trans* isomer, ~6.10 ppm) and C (*cis* isomer, ~5.76 ppm) used to determine isomer percentage. **c** Kinetics of AAB photoisomerisation studied by photo-NMR spectroscopy, with sample pre-equilibrated with blue light for 5 min before recording, and different colours toggled as indicated. The experimental data was fitted to mono- (black lines) and bi-exponential (blue lines) equations to extract kinetic parameters for individual transitions.

Photo-NMR may also be used to study reactions or transitions triggered by various colours of light, and is particularly well suited to studying the kinetics of such processes. The *trans*–*cis* isomerisation of azobenzene and its derivatives is one such light-induced transition, which may serve as a useful photoswitch in biotechnology applications^42^. Although some transitions in such system have been characterised by NMR before with in situ illumination^43–46^, exploring transitions triggered by toggling between numerous illumination colours would be more informative. To illustrate the application of the NMRtorch approach to characterise the kinetics of photoswitches, the photoisomerisation of 1 mM 4-aminoazobenzene (AAB) in DMSO-d6 was studied under continuous illumination with a variety of light wavelengths (Fig. 6). At equilibrium under darkness (Fig. 6b), AAB exists entirely as the *trans* isomer. However, if the solution is illuminated with short-wavelength light, such as blue, then isomerisation occurs, shifting equilibrium towards the *cis* isomer state, giving rise to distinct upfield NMR signals (Fig. 6b, marked with asterisks). Therefore, the ratio of these distinct signals can be used to derive populations of *trans* and *cis* isomers present in the sample, and track the kinetics of photoisomerisation in response to illumination by different light colours (Fig. 6c). Toggling between colours is easily achieved using a multi-channel NMRtorch lighthead containing four different LEDs (red, green, blue, white, RGBW).

The light toggling experiments reveal that, irrespective of the initial equilibrium state, *trans* → *cis* isomerisation triggered by blue light is fast, with rate constant *k* = 65 ± 6 µM min^−1^. Illumination with each colour establishes its own characteristic *trans*–*cis* equilibrium. After blue light illumination, re-equilibration towards *trans* is faster with green light (108 ± 3 µM min^−1^) than with red (*k* = 9.3 ± 0.1 µM min^−1^), but much higher equilibrium population of *trans* is reached with the red light than with the green. *Cis* → *trans* photoisomerisation under green and white light (111 ± 5 µM min^−1^) occurs at similar rates, but result in different ultimate equilibria, with more *trans* isomer present under green light. It should be noted that in this LED white light is generated by exciting luminophore with blue light, and therefore a strong blue component is present in this white light. Interestingly, unlike the colour-driven transitions which here all show mono-exponential behaviours, the thermal transition under darkness from *cis* to *trans* can be only satisfactory fitted as the sum of two exponents (*k*_fast_ = 17.9 ± 0.9 µM min^−1^, *k*_slow_ = 3.5 ± 0.1 µM min^−1^), highlighting the hidden complexity of this process uncovered by in situ photo-NMR experiments performed using NMRtorch. To our knowledge, the multi-stage mechanism of thermal transition under darkness for AAB has not been described before. One can also easily explore how the combination of colours would affect both the isomerisation rate and equilibrium state, and thus comprehensively characterise the behaviour of photoswitchable systems in response to different combinations of light stimuli and their intensities. The experiments here demonstrate the convenience of using NMRtorch and the potential of the multi-wavelength illumination approach in studying the kinetics of photoswitches, as well as other photo-induced phenomena, which can be detected and monitored in real time by NMR spectral changes.

## Discussion

In situ illumination of samples opens up a realm of potential light-sensitive experimental systems which may be studied by high-resolution NMR spectroscopy, one of the most powerful analytical techniques. Although a range of illumination strategies have been previously suggested, including guiding external light sources with optical fibres^29,30,47^, or modifying the NMR probe itself^20,21,27,34^, these solutions are generally cumbersome to use at higher throughputs, or require customised probes. Here, we present a new universal and convenient approach to illuminating NMR samples in situ with the LED-based NMRtorch device, which should be compatible with any typical NMR probehead, including cryoprobes. The lack of optical fibres minimises light losses and removes sources of magnetic field inhomogeneity, while allowing sample tube filling and capping to be done in the conventional manner, making photo-NMR experiments more user friendly and allowing higher throughput. Relatively large size of the LED-tube coupling interface allows positioning of several LEDs at once (here, up to four) close to each other and in close proximity to the tube rim, making possible experiments with multi-wavelength illumination. One can envisage that the proposed principles can be used in automation.

In the NMRtorch approach, the lighthead containing LEDs is attached directly at the top of a special NMR sample tube, and together they are inserted into the spectrometer, enabling illumination at a wide range of wavelengths, including UV, with a minimal light transmission path length. The NMRtorch tube itself acts as a light guide, with etched light-scattering patterns resulting in preferential illumination of the sample volume from the outside. Here, the position and extent of the etching used to introduce the light-scattering centres was observed to have a dramatic effect on the uniformity of light distribution and the resulting photo-CIDNP NMR signal (Fig. 3), in agreement with previous observations of the effect of roughening the exposed tip of fibre optics^29,30^. In our experience, both borosilicate and quartz NMRtorch tubes work well, although quartz tubes may be preferable at shorter wavelengths such as UV. As the typical light-emitting area of LEDs is comparable with the area of the transparent rim of the heavy-walled 5 mm NMRtorch sample tube, and the LEDs are in extremely close proximity to the rim, light coupling efficiency is very high even in the absence of additional optical elements such as condenser lenses. Local heating of this coupling area is not propagated to the sample area, and any local weak electromagnetic fields associated with LED switching are largely contained inside the shielded lighthead, also distanced from the NMR probehead. NMRtorch lightheads are generally cooled by the gas flow typically present in the magnet bore, and for more powerful LEDs, can be cooled further by the auxiliary compressed gas line.

In our experience, NMRtorch did not interfere with performance of the NMR spectrometer, with no noticeable effects on the signal lineshape, probe tuning or shimming, or solvent suppression. No additional re-equilibration delays were needed after light switching before the start of acquisition. NMRtorch sample tubes can be filled and sealed with caps as usual, allowing to work with oxygen- or moisture-sensitive samples, though transparent or semi-transparent tube caps are required to allow enough light to enter into the rim of the tube. As the walls of the tube need to be sufficiently thick for effective propagation of light and to achieve uniform light-scattering around the sample volume, the sample volume is therefore decreased to ~170 to 200 µL for a 5 mm tube, which is roughly equivalent to a standard 3 mm NMR tube. As the sample volume is illuminated effectively all round from the outside through the glass wall, radial distribution of light inside is more uniform than when a stripped optical fibre is used for the illumination from the inside: in the latter case, light diverges with the distance from the fibre surface (Supplementary Fig. 2). For NMRtorch, the light-scattering surface essentially functions as a diffuse extended (infinite planar) source of light wrapped around the sample area, where light intensity no longer depends on the distance from the source. Some light decay towards the centre of the sample would only be observed for light-absorbing samples. Overall, light losses are reduced, enabling significantly higher illumination intensity with the NMRtorch setup compared to an optical fibre-based setup with the same LED light source (Supplementary Fig. 7). NMRtorch enables experiments with optically dense liquid samples or suspensions, with effective optical path through the sample of less than ~1.2 mm. Importantly, no other materials, except the glass sample tube and the sample, are present in the proximity of the detection area, ensuring that magnetic field homogeneity is only limited by the quality of the glass tube and the sample itself. Importantly, NMRtorch setup is expected to be compatible with any standard high-field, low-field or benchtop NMR spectrometer, or probehead, and can be adapted to suit tubes of different diameters as well.

We trialled many different lighthead designs and specific arrangements of how LEDs are mounted inside, inspired by a wide variety of different flashlights available on the consumer market, and driven by an enormous variety of LED types and mounting footprints, and all these different designs worked sufficiently well (Supplementary Fig. 1). While lightheads containing single wavelength LEDs, or more standard combinations of LEDs (e.g., multi-colour RGBW) available as pre-mounted arrays, are easier to design and produce, custom combinations of assorted LEDs makes in-house manufacturing challenging. We note that, at the moment, the required components for building the lightheads are not commercially available, including elements for the lighthead housing and the metal printed circuit boards (MPCBs) where the LEDs are to be soldered, necessitating building and adapting of all these components from scratch. Industrial unified design and professional manufacturing of the lightheads should resolve this problem. Meanwhile, depending on the required application, combination of wavelengths, total light power required, desired LED types, spectrometer geometry, bore and tube size, and parts available, slightly different designs for in-house implementations can be anticipated and these will likely work as long as the main principle outlined in this work is followed – that light from closely positioned LEDs should enter the rim of the tube. Those wishing to try a do-it-yourself approach are encouraged to refer to the relevant LED manufacturer’s datasheets, which should guide them through the design and mounting of customised MPCBs specific for their application and desired combination of LEDs. Here, as a proof of principle, NMRtorch tubes were produced from commercially available heavy-walled NMR tubes by manually abrasively etching their outer surface, using diamond files, abrasive disks and/or sandpaper until the required light uniformity was achieved, using an approach which is very similar to manual roughening of the stripped tips of optical fibres commonly used in photo-NMR^11^. However, we also note that ultimately etching of the tubes using industrial machining methods is likely preferable to provide a supply of consistently made NMRtorch tubes as consumables.

Here, the NMRtorch demonstrated effective performance in typical photo-NMR experiments, including photo-CIDNP, studies of azobenzene photoisomerisation, and UV-induced chemical conversion. The 64-fold CIDNP enhancement observed here for 6FI with just one 3 W 460 nm LED is, to our knowledge, the highest observed in ^19^F NMR spectroscopy, either with LEDs or LASERs. This molecule, 6FI, has not been previously described as photo-CIDNP active, and its properties are well suited for assessment of light intensity distribution inside the samples, given that such enhancement is observed in the absence of any measures to remove oxygen or prevent dye quenching in the samples. For photo-CIDNP studies, we found LED PWM control directly by the NMR console to be particularly useful and convenient, and it removes the need for additional PWM light dimming electronic components as used elsewhere^46^. The multiple channel/LED aspect of the NMRtorch approach is also particularly advantageous for studies of photoswitches. One reversible photoswitch, DAE, was used here as a convenient actinometer system, taking advantage of the ability to pre-set different concentrations of its closed form using 280 nm UV LED, to enable light intensity measurements with visible light using a single sample (Fig. 4). Another photoswitch characterised here was azobenzene. Although photoisomerisation of azobenzene-based dyes has previously been studied by NMR spectroscopy using both in^43–46^ and ex situ^48^ illumination, rapid multi-colour switching of LED illumination demonstrated here enables additional kinetic and equilibria processes to be easily observed at a number of wavelengths, reducing the dead time of the experiments, and allowing to characterise fully the behaviour of a photoswitch in response to a range of stimuli (Fig. 6). Here, previously non-described bi-exponential thermal *cis* → t*rans* relaxation process of AAB in darkness has been revealed, suggesting a two-step relaxation mechanism, whereas all the light-induced transitions were mono-exponential. Finally, the NMRtorch also enables the use of very powerful LED arrays for sample illumination. Thus, photodegradation studies, normally conducted in dedicated UV illumination chambers, can be combined with online NMR spectral monitoring and analysis (Fig. 5). As critical light-conducting components of NMRtorch are made of glass, with relatively large cross-sectional area, no local degradation (e.g., yellowing) of these is expected following prolonged UV exposure.

In conclusion, the modular NMRtorch approach is capable of supporting combinations of multiple LEDs with different wavelengths, with detection using any NMR pulse sequence where the light control (i.e., LED channel, duration and intensity) commands can be added. As more powerful LEDs, laser diodes, or similar-sized new sources of illumination become available^49^, NMRtorch lightheads should be able to accommodate these and be used for an ever-increasing range of photo-NMR applications. NMRtorch can be used on all typical existing spectrometers and probes for any nuclei without any probe modification, opening new avenues of research in light-dependent phenomena by one of the most powerful analytical techniques.

## Methods

### NMRtorch apparatus design principles

The NMRtorch insert apparatus consists of: (i) a special light-conducting NMR sample tube; (ii) a lighthead which houses LEDs, positions them at the top of the NMRtorch tube and provides cooling for them; (iii) a power supply module; and (iv) connecting electric cables. Non-magnetic LEDs or LED arrays from different manufacturers were employed here, showing marginal difference in performance, with nominal emission wavelength and power as indicated for specific applications. A range of one- and four-channel power supplies to drive LEDs at constant currents, as per individual LED specifications, with transistor-transistor logic (TTL) trigger controls, were assembled in-house, as well as a number of lightheads of different designs with different combinations of LEDs (Supplementary Fig. 1). Heavy-walled 5 mm borosilicate (Wilmad/Hilgenberg) and quartz glass (Norell) NMR tubes were adapted by etching patterns on the exterior surface of the tubes around the bottom 40–42 mm area where the standard-size sample is located, thus making NMRtorch tubes. The etching was achieved in this instance by abrasion, using thin disks from hand tools, diamond files, or sandpaper of different grades, while the tube was rotated around its long axis. In the first instance, the success of etchings and uniformity of diffused light can be inspected visually by shining light from any readily available LED light source to the butt of the tube. We found that as long as LED is close, and the light enters the rim of the tube, satisfactory results can be obtained via many different designs; the emitter surface can be shifted from the axis of the tube by few millimetres without significantly compromising the light intensity, making it possible to mount several LEDs near the rim of the tube simultaneously. The way the LEDs were mounted was not critical, different approaches all led to similar satisfactory results. Similarly, use of lenses were trialled but these were of limited benefit, as LEDs usually could be positioned close enough for efficient light transfer. A range of transparent and semi-transparent tube caps from different materials were trialled, including use of sticky silicon dots, semi-transparent polyethylene tube caps, TEF film covering the top of the NMRtorch tube and held by a piece of PTFE tubing with 5 mm inner diameter, or using short pieces of hand-polished quartz 5 mm diameter glass rods sealed at the top of the NMR tubes using PTFE or PE tubing. However, we did not find the characteristics of this component critical for the experiments, as long as the cap tolerated the solvent used and local heating from the LED: all these makeshift caps worked in selected conditions.

### NMR spectroscopy

NMR experiments were performed on a Bruker 500 MHz Avance III spectrometer using a QCI-F cryoprobe with cooled ^1^H and ^19^F channels and sample temperature control unit. Spectra were initially processed and analysed using Topspin 4.1 and Dynamics Centre 2.7 (both Bruker), and plotted in GraphPad Prism 9.

### Photo-CIDNP

6-fluoroindole (6FI, Fluorochem) was prepared as a 250 mM stock solution in deuterated dimethylsulfoxide (99.8% DMSO-d6, Eurisotop), and diluted as required with H_2_O and 10% ^2^H_2_O (Sigma-Aldrich). Riboflavin 5’-monophosphate sodium salt hydrate (FMN, Sigma-Aldrich) was prepared as concentrated 10 mM stock, and added to the final samples immediately before experiments. No attempts were made to degas samples, or remove oxygen. The samples were placed in NMRtorch tubes and sealed with the transparent caps. The photo-CIDNP enhancement factor α has been calculated as:

1 $$\alpha =\frac{\left|{I}_{L}\right|}{{I}_{D}}$$

where *I*_*L*_ and *I*_*D*_ is the signal intensities in the illuminated and in the dark state, respectively. Light intensity (photosynthetic photon flux density, PPFD) at the exterior surface of the tube just outside the sample volume was measured with a calibrated Li-250A photometer and Li-190R quantum sensor (both Li-Cor).

### Measuring of light distribution in NMRtorch tubes

Pixel brightness images of selected example NMRtorch tubes with different patterns and degrees of etching were captured with a Pixel 4A (Google), and analysed for greyscale values in ImageJ software. Saturated fluorescein (Fluka) solution was prepared in water. Photo-CIDNP NMR imaging experiments were acquired with standard *zg* pulse sequence with ^19^F detection, modified to include variable light illumination before the 90° pulse, and application of a 0.106 G cm^−1^ magnetic field gradient (${G}_{z}$) during FID acquisition, typically with 4 scans. Dark spectra were recorded with the same pulse sequence beforehand without illumination with a sufficient number of scans (typically 16) to obtain the reference imaging profile for a given sample. In these imaging experiments, signals are offset in frequency ($\Omega$) according to:

2 $$\Omega =\gamma {G}_{{{{{{\rm{Z}}}}}}}Z$$

where $\gamma$ is gyromagnetic ratio, and *Z* is vertical position relative to the coil centre. To examine light distribution along the vertical *Z*-axis of the sample and remove the effect of gradient non-linearity at the edges, enhancement factors (${\alpha }_{{{{{{\rm{Z}}}}}}}$) at each point in the imaging spectra were calculated and related to the vertical position *Z* in the sample:

3 $${\alpha }_{{{{{{\rm{Z}}}}}}}\,=\frac{\left|{I}_{{{{{{\rm{L}}}}}}}^{\Omega }\right|}{{I}_{{{{{{\rm{D}}}}}}}^{\Omega }}$$

where ${I}_{{{{{{\rm{L}}}}}}}^{\Omega }$ and ${I}_{{{{{{\rm{D}}}}}}}^{\Omega }$ are profile intensities (normalised by the number of scans) in the illuminated and reference dark spectra at frequency offset $\Omega$, respectively. The values of ${\alpha }_{{{{{{\rm{Z}}}}}}}$ were used as a measure of local light intensity across the NMR-observable area of the sample.

### In situ actinometry

Intensity of light reaching the sample in the NMRtorch tube was assessed by monitoring the reversible closed form (CF) to open form (OF) photocyclisation of the diarylethene 1,2-bis(2,4-dimethyl-5-phenyl-3-thienyl)perfluorocyclopentene^36–38^ (DAE). DAE was prepared at 5 mM in dichloromethane-d_2_ and irradiated in situ using a lighthead containing 280 nm and 550 nm LEDs (nominal 3 W power consumption, driven at 0.7 A current each). Increasing concentrations of CF were obtained by illuminating with 280 nm light for variable periods of time, while 550 nm illumination was used to convert CF back to the OF and quantify the light intensity. During illumination, single scan NMR spectra were recorded at 5 s intervals to monitor the photoreaction. Experiments were repeated with both ^1^H and ^19^F NMR detection, with characteristic signal integrals used to determine isomer concentrations and linear fits used to determine the rate of CF conversion under 550 nm illumination for a given initial CF concentration. These rates were fitted to the following equation, adapted from the approach of Ji et al.^39^

4 $$-\frac{d[{{{{{{\mathrm{CF}}}}}}}]}{{dt}}={I}_{0}\Phi (1-{10}^{-\varepsilon b[{{{{{{\mathrm{CF}}}}}}}]})$$

where ${I}_{0}$ is light intensity, *Φ* is quantum yield, *ε* is molar absorptivity and *b* is the path length (see Supplementary Methods for further details). For the purposes of estimating light intensity, the *Φ* of CF → OF conversion was taken as 0.02 mol  Einstein^−1^ ^36,37^. Additionally, light intensity (photon flux density, PFD) at the exterior surface of the tube just outside the sample volume was measured with a calibrated PG200N spectral PAR meter (UPRtek).

### Azobenzene photoisomerisation

1 mM 4-aminoazobenzene (AAB, Fluorochem) was prepared in deuterated dimethylsulfoxide (99.8% DMSO-d6, Eurisotop). In situ NMR light illumination was performed with a lighthead containing red, green, blue and white LED array driven at 3 W (0.7 A current) per colour channel. As sample preparation under ambient light may lead to a poorly defined initial isomerisation state, the sample was pre-equilibrated in situ with blue light for 5 min before the start of acquisition. ^1^H NMR kinetic experiments were recorded at 5 s intervals with a pseudo-2D *zgesgp* pulse sequence, with 1 scan and 0 dummy scans, with the initial four spectra acquired in the series discarded to allow for magnetisation equilibration.

### Quinine photodegradation

2% (w/v) quinine hydrochloride dihydrate (Sigma-Aldrich) was prepared fresh as required in Milli-Q water with 10% ^2^H_2_O (Sigma-Aldrich). Quinine is the standard calibration material for UV photostability testing, as per International Council for Harmonisation of Technical Requirements for Pharmaceuticals for Human Use (ICH) Q1B guidelines^40,41^. In situ illumination was performed with a UV LED array (nominal 365 nm peak emission, 10 W power), in NMRtorch tubes made of either borosilicate glass or quartz. UV–Vis spectra for control (foil-wrapped to protect from light and stored at the same temperature for the duration of the experiment) and illuminated samples were recorded using a Nanodrop 2000 (ThermoScientific).

## Supplementary information

Supplementary Information

Description of Additional Supplementary Files

Supplementary Software 1

## Data Availability

All data generated or analysed during this study are included in this published article (and its Supplementary Information). Pulse programme for PWM control of illumination brightness available as Supplementary Software 1.
