# Supplementary material for: Sample illumination device facilitates in situ light-coupled NMR spectroscopy without fibre optics
Source: Commun Chem. 2022 Aug 4;5:90. doi: 10.1038/s42004-022-00704-5 (PMC9814378; doi:10.1038/s42004-022-00704-5)
Supplement: Supplementary file 1 — Supplementary Information [file 42004_2022_704_MOESM1_ESM.pdf]

Supplementary Information

Sample illumination device facilitates in situ light-coupled NMR spectroscopy without fibre optics

Jack E. Bramham[1] and Alexander P. Golovanov[1*]

[1] Department of Chemistry, School of Natural Sciences, Faculty of Science and Engineering, The University of Manchester, Manchester, M13 9PL, United Kingdom

* Send Correspondence to a.golovanov@manchester.ac.uk

Table of Contents:

Supplementary Methods. (**S-2**)

Supplementary Figure 1: Examples of prototype NMRtorch hardware used for sample illumination. (**S-4**)

Supplementary Figure 2: Schematics of radial light distribution in a sample tube containing optical fibre with roughened light-scattering surface and NMRtorch tube. (**S-5**)

Supplementary Figure 3. Example of an NMRtorch tube with uniform light distribution as judged by NMR imaging experiments. (**S-6**)

Supplementary Figure 4: Evolution of DAE open (OF) and closed form (CF) characteristic NMR signals under UV and lime light illumination. (**S-7**)

Supplementary Figure 5: Online 1D ^{1}H NMR assessment of quinine photodegradation in a borosilicate NMRtorch tube with *in situ* illumination using 10 W LED array with peak emission at 365 nm. (**S-8**)

Supplementary Figure 6: Degradation of quinine after 2 hours UV irradiation in a quartz NMRtorch tube using 10 W LED array with peak emission at 365 nm. (**S-8**)

Supplementary Figure 7: Comparison of light intensity for NMRtorch and optical fibre setups. (**S-9**)

Supplementary References. (**S-10**)

Supplementary Methods

Quantification of light intensity using NMR actinometry

The intensity of light delivered to the NMR sample was measured using the reversible diarylethene-based actinometer DAE (1,2-bis(2,4-dimethyl-5-phenyl-3-thienyl)perfluorocyclopentene).[1-3] Here, the rates of CF→OF conversion by visible light were fitted to the following equation using the approach of Ji, et al. [4]:

$$-\frac{d[CF]}{dt} = I_0\Phi(1 - 10^{-\varepsilon b[CF]})$$

where I_0 is the light intensity in the sample, Φ is quantum yield, ε is molar absorption coefficient, and b is the path length. Here, for the purposes of estimating light intensity, the Φ of CF→OF conversion by 550 nm visible light was taken as 0.02 mol Einstein^{-1}.[1-3] Path length (from the inner wall of the NMRtorch tube to the centre of the sample) for the modified heavy wall quartz tube (Norell) used was determined as 0.116 cm. By simultaneously fitting to ^{1}H and ^{19}F NMR derived photocyclisation rates (Figure 4e), I_0 was determined as 438 ± 21 µEinstein L^{-1} s^{-1}, while ε was 15500 M^{-1} cm^{-1} (95% confidence interval = 12700 to 19300 M^{-1} cm^{-1}). The value of ε measured here in dichloromethane is in reasonable agreement with value of 10900 M^{-1} cm^{-1} reported previously based on UV-Vis measurements of HPLC-purified DAE CF in n-hexane or ethanol.[1,3] We note that for the latter measurements any CF→OF conversion occurring due to unavoidable illumination during the UV-Vis measurements themselves may distort the actual CF concentration, resulting in underestimation of the absorption coefficient. This may also explain the minor difference with the ε obtained using our approach, where the measurements of starting CF concentrations *in situ* by NMR (which do not rely on optical detection) are expected to be inherently more robust.

The value of I_0 (438 µEinstein L^{-1} s^{-1}) reported above provides an estimate of the total number of photons reaching the sample per unit volume per second. Knowing that the sample forms a cylinder with known dimensions (4.0 cm length and 0.232 cm diameter) and known volume (169 µL), and that light enters the sample uniformly through the entire surface of this cylinder, it is possible to calculate the photon flux through the sample surface. The photon flux per sample volume (0.079 µmol s^{-1}) was converted to photon flux density through the surface of the sample, which for the sample surface area of 2.96 cm^{2} yields a photon surface flux density of 251 µmol m^{-2}

s^{-1}. Conversely, the flux density measured on the exterior of the light scattering area of the tube with a spectrophotometer was 143 µmol m^{-2} s^{-1}, which is noticeably smaller. This reduced measured flux can be explained by the fact that light diverges outwards from the tube surface and photon flux reduces with distance, while the flat spectrophotometer probe does not conform precisely to the surface of the NMR tube, creating a gap between them. We therefore conclude that the measurements taken with a photometer on the tube exterior provide a rough lower estimate of the amount of light reaching the sample. As a reference, according to measurements using the photometer, the photon flux density of 251 µmol m^{-2} s^{-1} illuminating the sample in the NMRtorch tube is equivalent to the sample being spread evenly over 2.96 cm^2 at a depth of 0.116 cm and directly illuminated with the same LED from a distance of ~5 cm.

Supplementary Figures

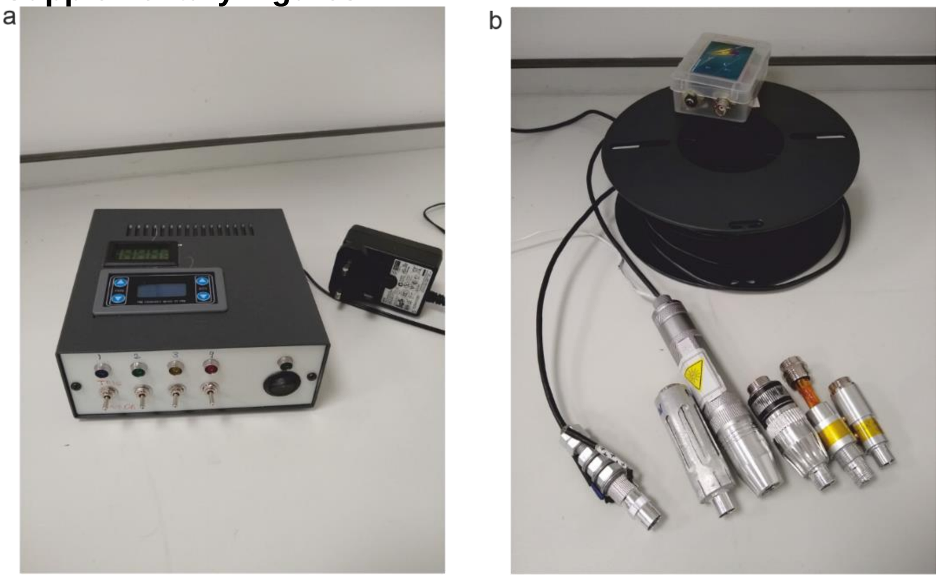

Supplementary Figure 1. Examples of prototype NMRtorch hardware used for sample illumination. (**a**) The four-channel power supply, with PWM LED dimming feature and temperature monitor. (**b**) One-channel power supply (top) wired to the blue LED lighthead (bottom left) used for photo-CIDNP experiments. Second left is the RGBW (red-green-blue-white) lighthead used for aminoazobenzene photoswitching experiments. Other example lightheads, from left to right, are: 1-channel UV; 3-channel; 4-channel and 2 channel.

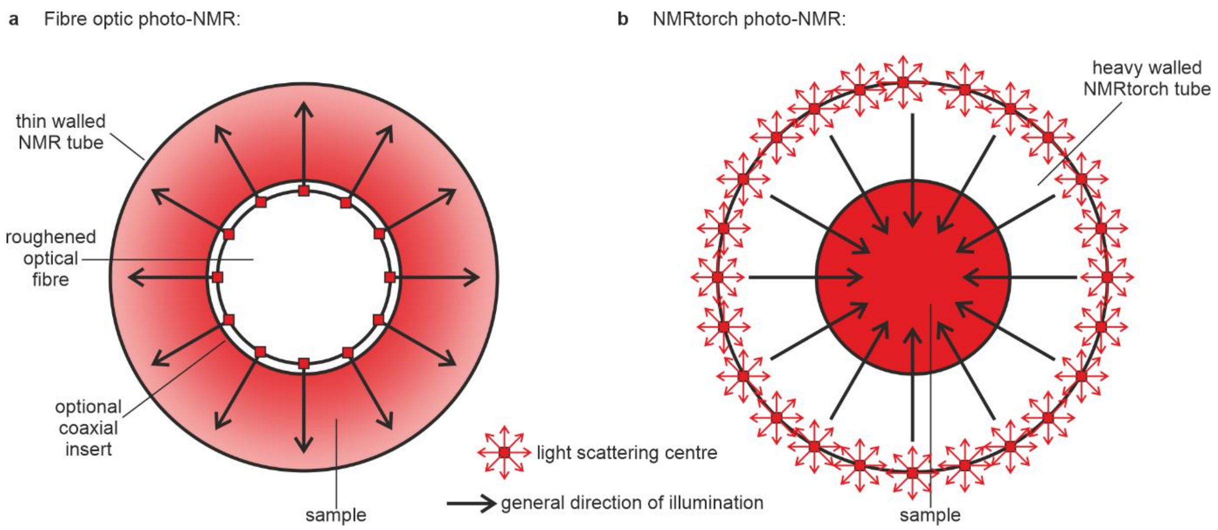

Supplementary Figure 2. Schematics of radial light distribution in sample tube containing optical fibre with roughened light-scattering surface (a) and NMRtorch sample tube (b). Cross-sections of the tubes are shown, with illuminated sample areas coloured red. In both fibre optic and NMRtorch setups, light scattering centres on the exterior surface of the optical fibre and NMRtorch tube, respectively, result in scattering of light and illumination of the sample volume. Here, for simplicity, we consider radial light distribution in a scenario where the sample itself does not absorb light. In the fibre optic setup (a), illumination diverges outwards from the fibre optic in the interior of the sample towards the exterior, spreading over larger area and thus decreasing here as *~1/r* where *r* is the distance from the centre. This results in the periphery of the sample experiencing lower light intensity (i.e., power output per unit area) than the interior of the sample closest to the optical fibre surface. Conversely, in the NMRtorch approach (b) the sample is illuminated from the exterior, with light converging inwards towards the centre of the sample from all directions, making radial distribution of light intensity essentially uniform. In the case of light-absorbing samples, there will be an additional contribution to non-uniformity for both cases, but with nearly identical effective optical path in (a) and (b) of around 1.1 mm, this contribution will be similar, suggesting that, under all conditions, overall radial light distribution in the NMRtorch setup is more uniform than for the optical fibre approach.

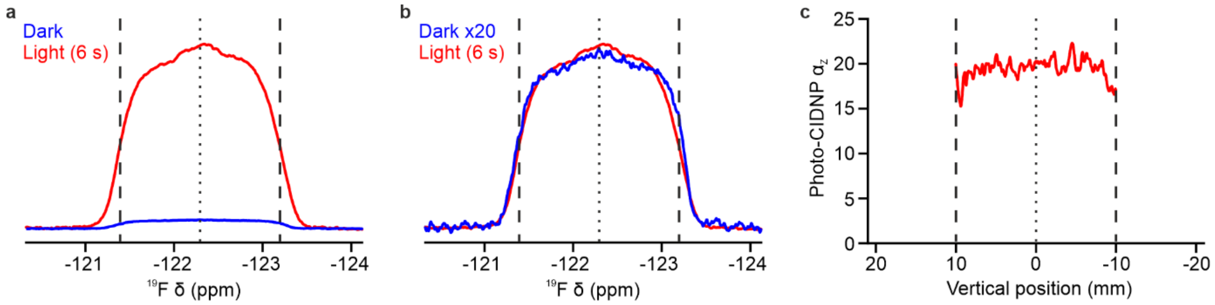

Supplementary Figure 3. Example of an NMRtorch tube with uniform light distribution as judged by NMR imaging experiments. (**a**) Raw NMR images in the dark (blue traces) or with 6 s illumination (red traces). (**b**) Dark NMR image scaled 20-fold to approximate CIDNP enhancement. (**c**) Position-dependent photo-CIDNP enhancement (α_Z) demonstrating reasonable linearity across the tube. Average α_z = 18.9 ± 1.1, corresponding to ~6% variation in uniformity of light intensity along the length of the sample. The sample was 6 mM 6FI with 0.2 mM FMN. Dashed lines indicate edges of the used imaging region at ±10 mm, while dotted lines indicate centre of sample volume.

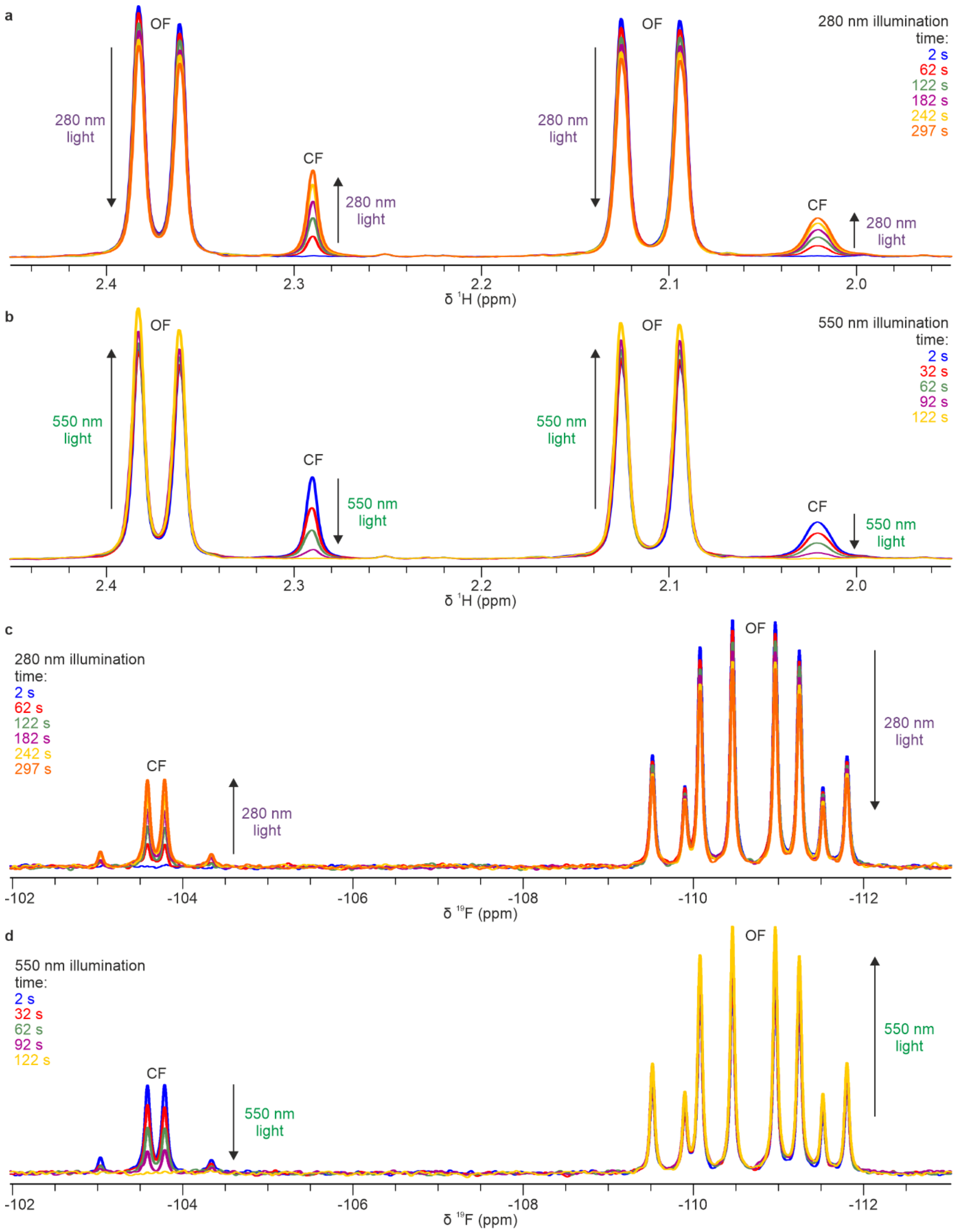

Supplementary Figure 4. Evolution of DAE open (OF) and closed form (CF) characteristic NMR signals under 280 (UV) and 550 nm (visible) light illumination. ^{1}H NMR spectra under (**a**) UV and (**b**) visible light illumination. ^{19}F NMR spectra under (**c**) UV and (**d**) visible light illumination. Spectra acquired under UV illumination plotted at 60 s intervals, while those under visible light illumination plotted at 30 s intervals.

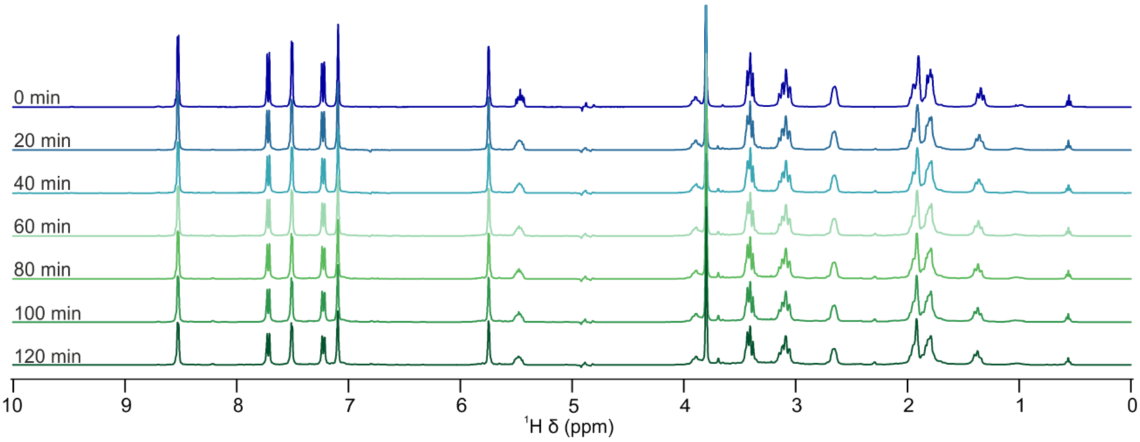

Supplementary Figure 5. Online 1D ^{1}H NMR assessment of quinine photodegradation in a borosilicate NMRtorch tube with *in situ* illumination using 10 W LED array with peak emission at 365 nm. Spectra were acquired throughout the course of illumination, with representative time points shown.

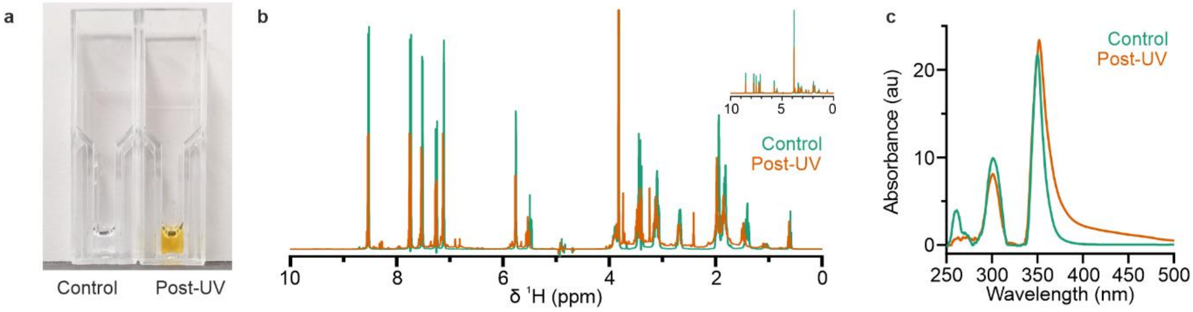

Supplementary Figure 6. Degradation of quinine after 2 hours UV irradiation in a quartz NMRtorch tube using 10 W LED array with peak emission at 365 nm. Comparison of quinine pre- and post-UV irradiation, as assessed by visual comparison (**a**), ^{1}H NMR spectroscopy (**b**), and UV-Vis spectroscopy, with absorbance change ΔA_{400} 2.12 ± 0.03 (**c**). The absorbance change far exceeds the minimum requirements of ICH Q1B guidelines.

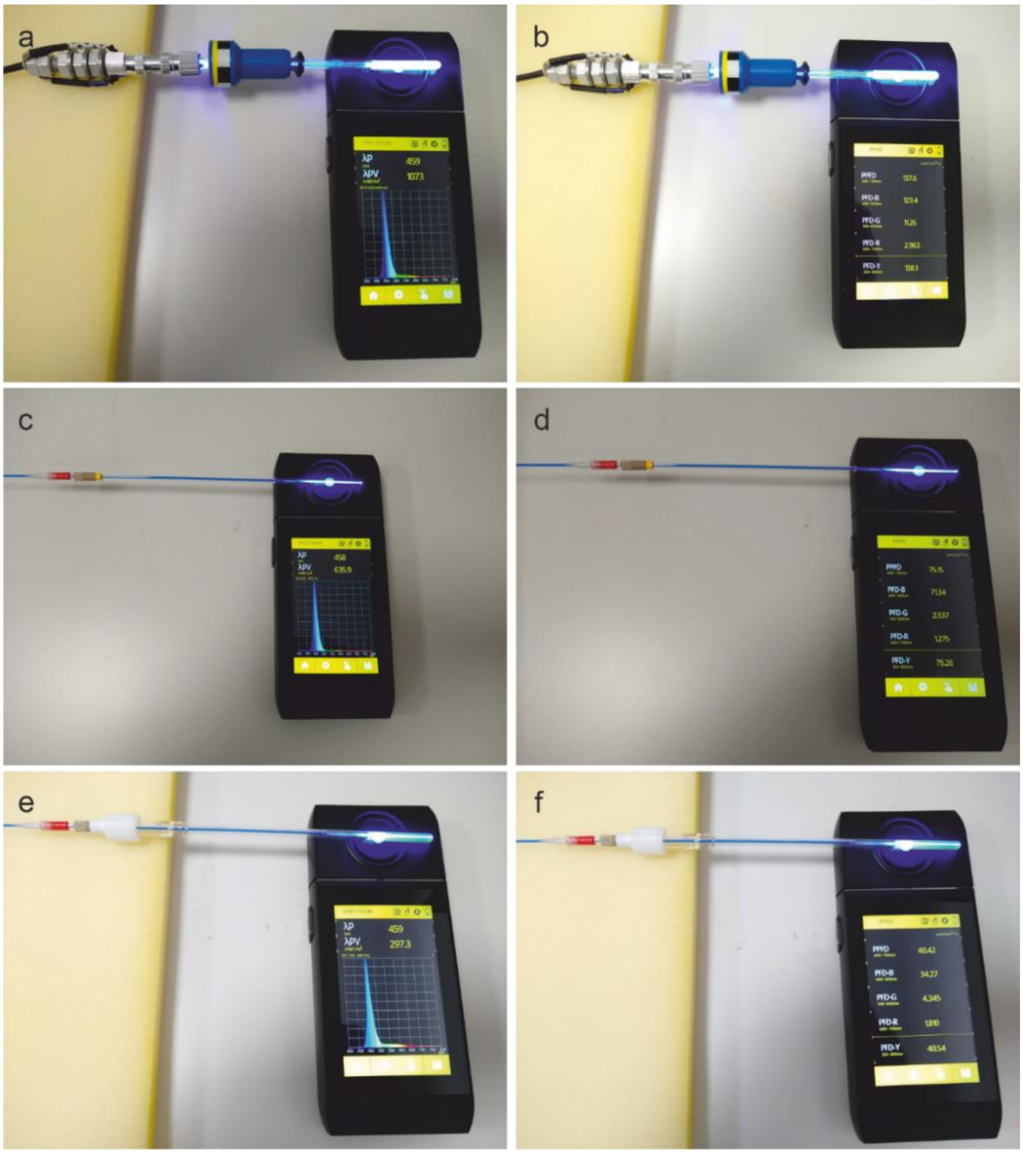

Supplementary Figure 7. Comparison of light intensity for NMRtorch and optical fibre setups measured just outside the sample area using photometer. The samples contain 6 mM 6FI and 0.2 mM FMN in 90% water/10% D_2O. The same LED (3 W, ~460 nm) was used for illumination, either as part the NMRtorch lighthead or butt coupled to the fibre. The fibre (Thorlabs) with 1 mm diameter was roughened at the stripped end and inserted in a New Era photoNMR sampling device tube through coaxial insert. For the NMRtorch setup, the measured light intensity on the exterior of the filled tube was 1073 mW m^{-2} (**a**) with PPFD of 138 µmol m^{-2} s^{-1} (**b**). For the optical fibre approach, light intensity just outside the insert itself was 636 mW m^{-2} (**c**) with PPFD of 75 µmol m^{-2} s^{-1} (**d**), while light intensity on the exterior of the filled NMR tube was 298 mW m^{-2} (**e**) with PPFD of 40 µmol m^{-2} s^{-1} (**f**). For the fibre optic approach, the measured light intensity on the exterior of the coaxial insert (c-d) provides an upper estimate of light intensity entering the sample. Conversely, for the NMRtorch approach, the measurement at the tube exterior (a-b) provides a lower estimate for light intensity, as the detector is positioned away from the illuminated sample. The measured background from ambient light illumination in the room was 4.9 mW m^{-2} with PPFD of 3.5 µmol m^{-2} s^{-1}, hence providing an insignificant contribution. The photometer is PG200N spectral PAR meter (UPRtek). For comparison, the photo-CIDNP enhancement obtained for the fibre-based configuration shown on panels (e,f) was only ~16-fold, compared to typical ~64-fold enhancement obtained using NMRtorch setup, panels (**a,b**). This suggests that when the optical fibre is used, the effective light intensity reaching the sample is around 4 times weaker, compared to NMRtorch.

Supplementary References

1 Sumi, T., Takagi, Y., Yagi, A., Morimoto, M. & Irie, M. Photoirradiation wavelength dependence of cycloreversion quantum yields of diarylethenes. *Chem Commun (Camb)* **50**, 3928-3930, doi:10.1039/c4cc00396a (2014).

2 Roibu, A. *et al.* An accessible visible-light actinometer for the determination of photon flux and optical pathlength in flow photo microreactors. *Sci Rep* **8**, 5421, doi:10.1038/s41598-018-23735-2 (2018).

3 Maafi, M. & Al-Qarni, M. A. Mono- and polychromatic light diarylethene-actinometer for the visible range. *Dyes and Pigments* **198**, 109942, doi:10.1016/j.dyepig.2021.109942 (2022).

4 Ji, Y., DiRocco, D. A., Hong, C. M., Wismer, M. K. & Reibarkh, M. Facile Quantum Yield Determination via NMR Actinometry. *Org Lett* **20**, 2156-2159, doi:10.1021/acs.orglett.8b00391 (2018).
