# Supplementary material for: Sample illumination device facilitates in situ light-coupled NMR spectroscopy without fibre optics
Source: Commun Chem. 2022 Aug 4;5:90. doi: 10.1038/s42004-022-00704-5 (PMC9814378; doi:10.1038/s42004-022-00704-5)
Supplement: Supplementary file 2 — Description of Additional Supplementary Files [file 42004_2022_704_MOESM2_ESM.docx]

Description of Additional Supplementary Files

**File name:** Supplementary Software 1

**Description:** 1D pulse sequence with pulse-width modulation control of illumination brightness
