# Supplementary material for: Sample illumination device facilitates in situ light-coupled NMR spectroscopy without fibre optics
Source: Commun Chem. 2022 Aug 4;5:90. doi: 10.1038/s42004-022-00704-5 (PMC9814378; doi:10.1038/s42004-022-00704-5)
Supplement: Supplementary file 3 — Supplementary Software 1 [file 42004_2022_704_MOESM3_ESM.pdf]

Bramham & Golovanov. *Sample illumination device facilitates in situ light-coupled NMR spectroscopy without fibre optics*. 2022

Supplementary Software 1: 1D zg pulse sequence with pulse-width modulation (PWM) control of illumination brightness

```
;1D zg sequence with LED illumination control by pulse-width
;modulation(PWM)before RF pulses
;05/05/21 Jack Bramham, Golovanov group, The University of Manchester
;
; set the value of cnst61 to required % of light brightness (0-100%)
; set the value of d30 for required total illumination time (seconds)
;
;$CLASS=HighRes
;$DIM=1D
;$TYPE=
;$SUBTYPE=
;$COMMENT=

#include <Avance.incl>

define loopcounter PWMCounter ;counter for PWM loops
define delay OnDelay ;duration of illumination during PWM cycles
define delay OffDelay ;duration of illumination off during PWM cycles

"acqt0=-p1*2/3.1416"
"l7=cnst61"
"OnDelay = (cnst61/100)*d31"
"OffDelay = d31-OnDelay"
"PWMCounter = d30 / d31"

1 ze
2 30m
  d1
if “d30==0” goto 4 ;no illumination delay = skip light loop entirely
if (l7==0) ;no light loop
{
  0.05u
  d30 ;delay with no illumination
  0.05u
  goto 4 ;skip light loops and go to pulse and acquisition
}
  if (l7==100) ;full brightness loop
{
  0.05u setnmr3|10 ;light turned on
  d30 ;light on during this delay
  0.05u setnmr3^10 ;light turned off
}
else ;pulse width modulation loop
{
  "OnDelay = (cnst61/100)*d31"
  "OffDelay = d31-OnDelay"
  "PWMCounter = d30 / d31"
3 0.05u setnmr3|10 ;light turned on
  OnDelay ;light on during this delay
  0.05u setnmr3^10 ;light turned off
  OffDelay ;light off during this delay
  lo to 3 times PWMCounter ;loop for PWM cycles
}
4
  d16
  p1 ph1
  go=2 ph31
  30m mc #0 to 2 F0(zd)
exit

ph1=0 2 2 0 1 3 3 1
ph31=0 2 2 0 1 3 3 1

;pl1 : f1 channel - power level for pulse (default)
;p1 : f1 channel -  high power pulse
;d1 : relaxation delay; 1-5 * T1
;d16 : post-illumination delay
;d30 : total illumination time
;d31 : PWM cycle time
;ns: 1 * n, total number of scans: NS * TD0
;cnst61 : PWM duty-cycle (0-100% brightness)
```
